# An account on the innovative path of cooperation with Prof. Zhiguo Su in bioseparation

**DOI:** 10.1002/elsc.202100016

**Published:** 2021-06-07

**Authors:** Jan‐Christer Janson

**Affiliations:** ^1^ Uppsala Biomedical Center Uppsala University Uppsala Sweden

When participating in the Asia Pacific Biochemical Engineering Conference (APBioChEC) in Singapore in 1994, I was fortunate to attend a workshop seminar given by Prof. Zhiguo Su from Dalian University of Technology, Dalian, PRC. The title of the seminar was “Bioseparation in the Peoples Republic of China” in which he displayed a profound knowledge of the current situation for biotechnology in his homeland. After the seminar I approached Prof. Su introducing myself. We immediately realized that we shared a strong interest in protein purification and the same year he invited me to give a lecture to his students and staff at his department.

Soon after, in 1997, Prof. Su was appointed Deputy Director of the State Key Laboratory of Biochemical Engineering in the Institute of Process Engineering at the Chinese Academy of Sciences in Beijing and promoted to its Director in 2001. In 2013 till date, Prof. Su has been Chief Scientist at the National Engineering Research Center for Biotechnology in Beijing. During all these years we have kept a close contact for joint research in the area of bioseparation.

Bioseparation is a key support area of research for the development of modern life science and technology. In scientific laboratories, biomolecules are separated to ensure a pure substance for further study. In industry, various biological products, such as blood proteins, vaccines and recombinant pharmaceuticals, have to be purified to guarantee safety, activity and long term stability. Despite its importance, not many researchers would like to engage in bioseparation research because it involves multidisciplinary knowledge and know‐how experience. Furthermore, it is not an area generating publications in journals of high impact factors.

Professor Su and I share a strong interest in bioseparation because we both realize its power in solving practical problems both in life science laboratories and in industry. For more than 20 years, we have collaborated on the path of innovation. Here, I would like to introduce some of our research results and corresponding techniques.

## 1 CHROMATOGRAPHIC REFOLDING OF PROTEINS

**FIGURE 1:**
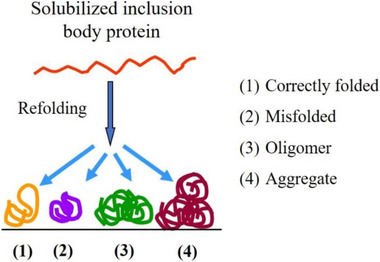
Possible products of refolding for solubilized inclusion body proteins

This area is very representative of our successful cooperation. In the production of genetically engineered proteins by overexpression in *E. coli*, it is often difficult to avoid the formation of inclusion bodies requiring the restoration the denatured protein to its natural active state. The formation of inclusion body protein has the advantage of high expression, short fermentation time and easy separation from other components. However, the inclusion body protein must be solubilized using strong denaturants such as guanidine hydrochloride or urea followed by refolding to its naturally active configuration. Misfolding and aggregate formations are unfortunately difficult to avoid (Figure 1). Dilution refolding, a common method in industry, brings about huge volume increase of processing stream and extremely low protein concentration.

Our idea was to use a solid medium to assist the protein folding. A solid medium might bind a protein through various interactions such as hydrophilic, hydrophobic, or physical structures. Proper control of these interactions would help the denatured protein fold to the correct conformation without misfolding. This idea reminded us of chromatographic media for protein purification. If the denatured proteins can be folded when bound to a solid media in a chromatographic process, it may be a useful combination of simultaneous protein refolding and separation. Thus, we carefully designed the following strategies for experimental approach:

1. Creating an environment in which the denaturant concentration decreased gradually in the chromatographic column to avoid molecular aggregation caused by a sudden change in the denaturant concentration. The strategy of the gradual change was designed based on the refolding thermodynamics and kinetics of the protein molecules. The process was performed using a computerized chromatographic workstation that made different solution environments possible.
2. Focusing on size exclusion, ion exchange and hydrophobic interaction because these are the most commonly used chromatographic processes for protein separation. The challenge would be the simultaneous kinetic control of refolding and separation. If successful, the refolding process could well be integrated into the current industrial process.
3. Starting with lysozyme, an easily characterized and well documented protein, as the model but aiming at solving the real problems of genetically engineered inclusion body proteins, such as granulocyte colony stimulating factor (GCSF), interferons, single chain antibody (scFV), non‐glycosylation erythropoietin (ngEPO).

**FIGURE 2:**
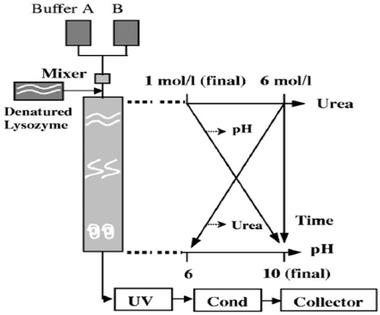
Concept of the protein refolding in size exclusion chromatography

Our cooperation achieved exciting results. The first approach was size exclusion chromatography (SEC) refolding. As shown in Figure 2, the urea concentration is allowed to decrease along the length of the SEC column from 6 to 1 M. Meanwhile, the pH is increased from 6 to 10. Denatured lysozyme in 6 M urea, pH 6, moves down the column and folds gradually to its original active configuration. Compared with dilution refolding, the SEC refolding increased the activity recovery from 30 to 80% at protein concentration of 30 mg/mL [1].

**FIGURE 3:**
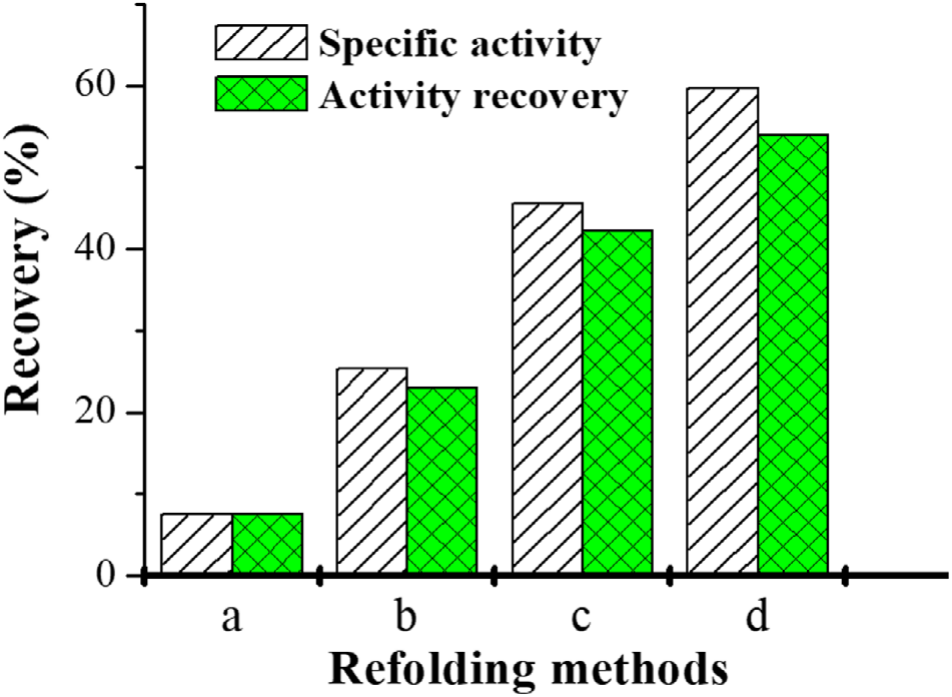
Comparison of scFV refolding by dilution and by SEC. (a) Dilution; (b) SEC without gradient; (c) SEC with denaturant gradient; (d) SEC with dual gradients of denaturant and pH

In Figure 3 is demonstrated the comparative results of refolding for single chain antibody scFv 57P. Clearly, column refolding with SEC increased the recovery greatly. Furthermore, dual gradient refolding with urea and pH presented the best recovery [2].

**FIGURE 4:**
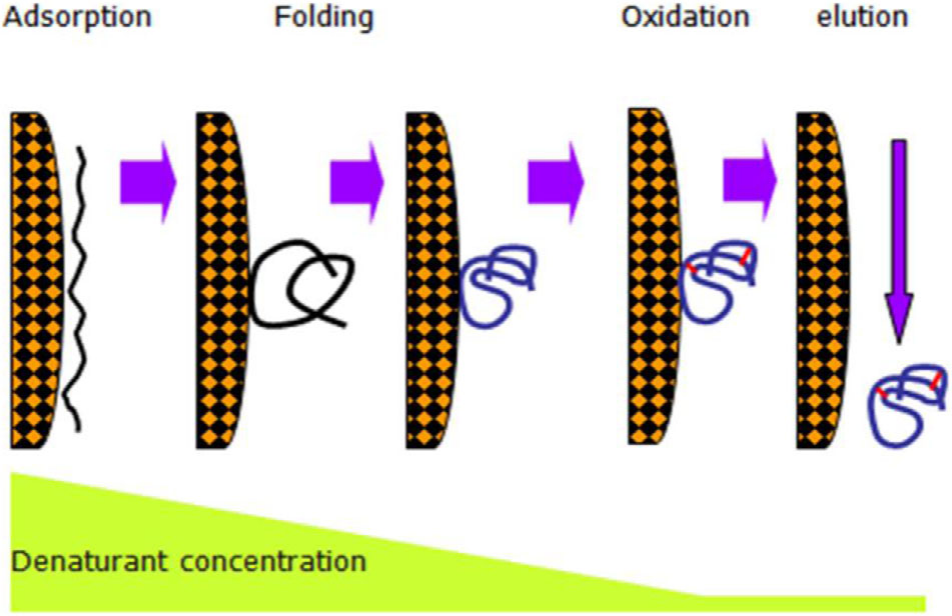
Concept of the protein refolding in ion exchange chromatography (IEC) or in hydrophobic interaction chromatography (HIC)

We then extended to ion exchange chromatography (IEC) [3] and hydrophobic interaction chromatography (HIC) [4]. The strategy is illustrated in Figure 4 where the decrease of the denaturant concentration initiates the refolding gradually with the denatured protein adsorbed to the solid matrix. The final oxidation step is designed for the formation of disulfide bonds within the molecule, which is necessary when refolding complex proteins. Table 1 shows a comparison of dilution refolding with IEC refolding for the renaturation of recombinant human lysozyme. Although the dilution took less time, its final concentration was very low, that is, 0.03 mg/mL. In contrast, the concentration of the IEC refolded protein was more than 20 times higher, reaching about 0.8 mg/mL [5].

**TABLE 1:** Comparison of two refolding methods for renaturation of recombinant human lysozyme

Item of comparison	Dilution refolding	IEC refolding
Process time (h)	<1	>2
Final protein concentration (mg/mL)	0.03	0.6
Protein recovery (%)	100	98
Activity recovery (%)	29	95
Specific activity (U/mg)	12,900	41,418

**FIGURE 5:**
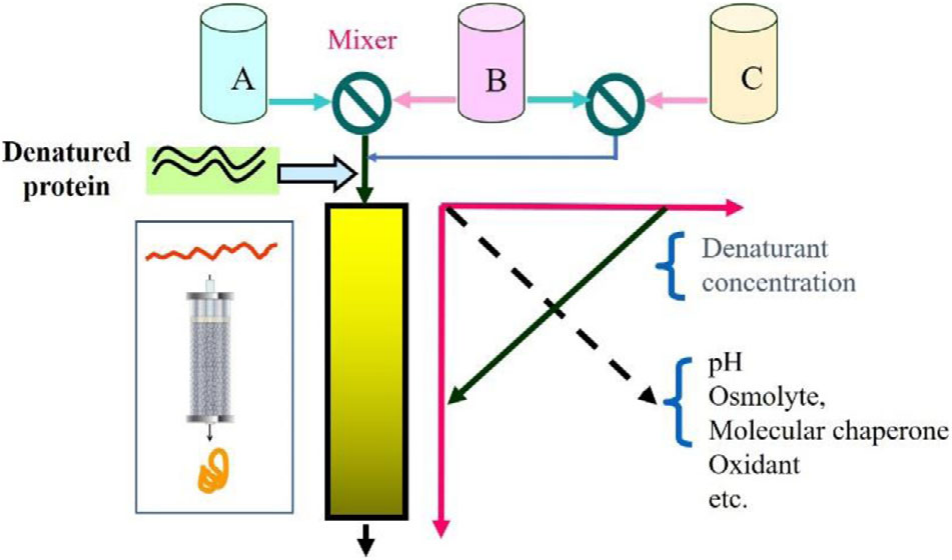
Generalized illustration for chromatographic refolding of proteins

Summarizing the results of the above three chromatographic processes, a common protocol can be developed as shown in Figure 5 in which the column could be the SEC, the IEC, the HIC, or affinity (AC). The concentration change (gradient) is not limited to pH or denaturants, but might be extended to various electrolytes, molecular chaperones and oxidants that are necessary for refolding a particular target [6].

Our approach was quickly adopted by other researchers who developed or improved new chromatographic refolding processes. The publications of our work were cited hundreds of times. One of them was awarded the Top 5 download paper in the journal *Protein Expression and Purification* in 2002. Pharmaceutical companies approached us for help to solve their refolding problems of recombinant proteins, such as human single‐chain antibodies, human lysozyme, Fe‐SOD, human colony stimulating factor, alpha‐interferon, *Staphylococcus aureus* prolongation factor G, etc. [7, 8].

## 2 UNIFORM‐SIZE CHROMATOGRAPHY MEDIA FOR HIGH‐RESOLUTION PROTEIN PURIFICATION

**FIGURE 6:**
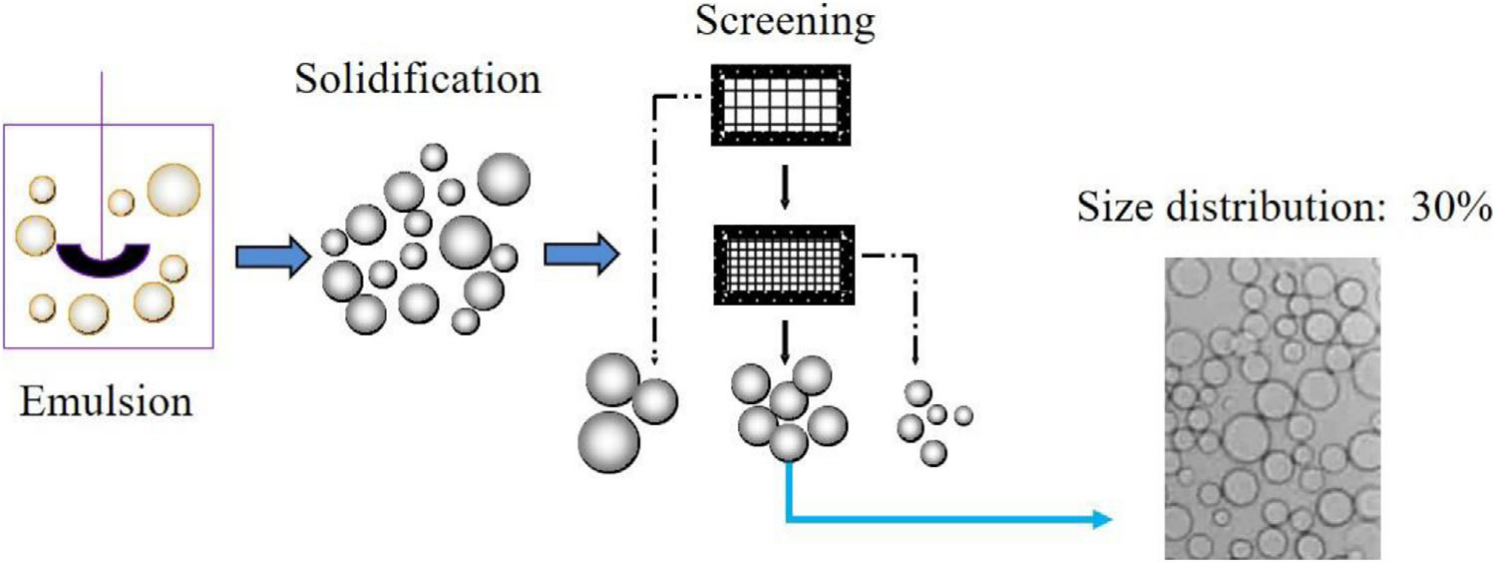
Conventional preparation of polysaccharide microspheres. The emulsion is prepared by mechanical stirring to disperse the polysaccharide solution in oil phase. Solidification is done by temperature change or chemical crosslinking

For a long time Professor Su and I have been interested in new efficient chromatographic purification media. Although microspheres based on the marine polysaccharide agarose have been widely used in protein purification since more than 50 years, they suffer from three disadvantages. The first is their polydispersity due to the fact that the industrial manufacturing process is based on mechanical stirring, resulting in wide size distributions (Figure 6). The second is that it is difficult to make high concentration agarose microspheres by mechanical stirring because the very high solution viscosity. The third is that it is difficult to prepare agarose microspheres smaller than 20 microns in size, which are required for high resolution separation, on an industrial scale.

In 2001, Professor Guanghui Ma joined the State Key Laboratory of Biochemical Engineering (SKLBCE) in China at the invitation of Prof. Su. Prof. Ma is a well‐known polymer microsphere expert. With her participation, we were able to establish a framework of chromatographic media innovation. The research went well at the SKLBCE because of the strong support from Prof. Su who is a well‐known leader in organizing a strong and excellent R&D team. Prof. Ma and Prof. Su invented an advanced technology of membrane emulsification to prepare homogeneous agarose polysaccharide microspheres.

**FIGURE 7:**
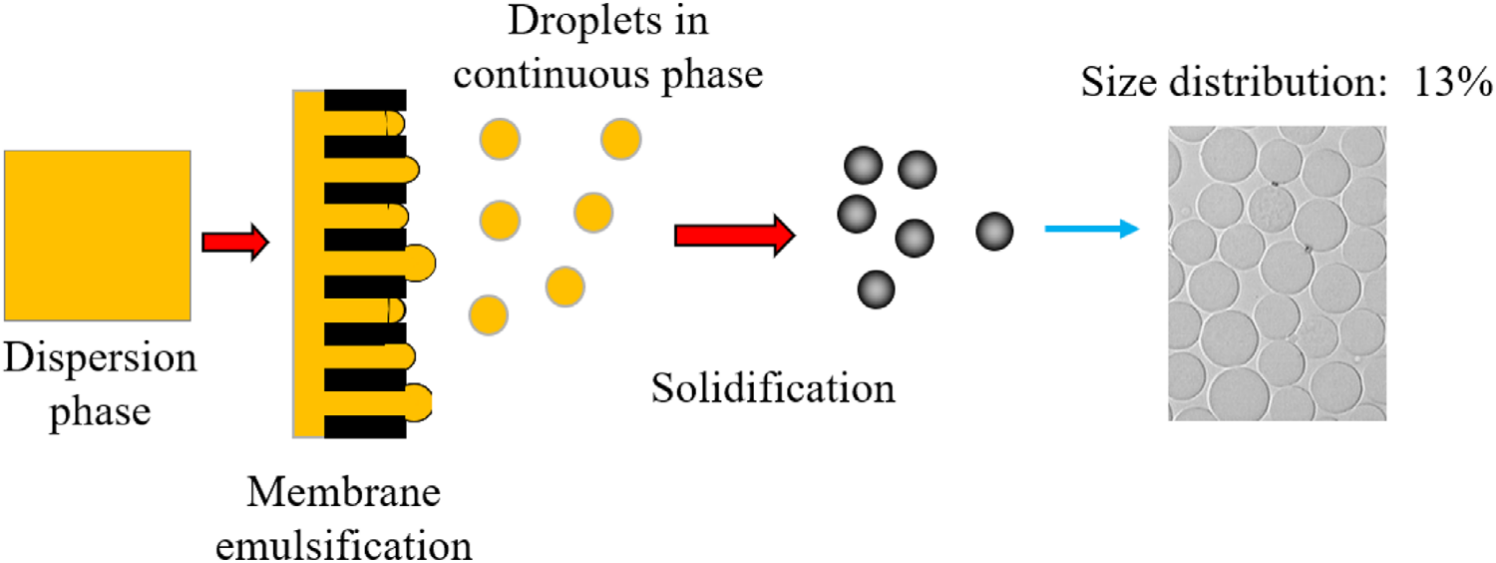
Novel preparation of polysaccharide microspheres by membrane emulsification. The dispersion phase is the polysaccharide solution that is going through a microporous membrane to generate uniform emulsion. Solidification is done by temperature change or chemical crosslinking

Their invention is shown in Figure 7 where a relatively uniform agarose polysaccharide emulsion is achieved after being forced through a microporous membrane [9]. The whole agarose polysaccharide solution is 100% converted to uniform microspheres without any waste and sieving. The particle size can be controlled by selection of membranes with different pore size. High concentration agarose polysaccharide microsphere packings can be prepared by their further invention of a dual membrane emulsification process [10] to ensure high mechanical strength, smaller particle size for high resolution separation, which are difficult or impossible by conventional preparation methods.

GE Healthcare Bio‐Sciences in Uppsala, Sweden (now Cytiva) is the world leader in the manufacturing of chromatography media and equipment for bioscience research and biotechnology industry. The scientific achievements by Prof. Ma and Prof. Su captured strong interest of the company who decided to sign a contract with the two professors and their institute for technology transfer of the membrane emulsification technique. The cooperation has been very successful. Thus, using the membrane emulsification technology, Cytiva are now manufacturing, world‐wide marketing and selling, uniform size agarose polysaccharide microspheres under the trade names *Superdex 200 Increase*, *Superdex 75 Increase*, *Superdex 30 Increase*, and *Superose 6 Increase*, as advanced chromatographic packings for the purification of peptides, proteins, antibodies, vaccines and various pharmaceuticals. It is the first time for this company to employ a Chinese invention in their product development and production.

**FIGURE 8:**
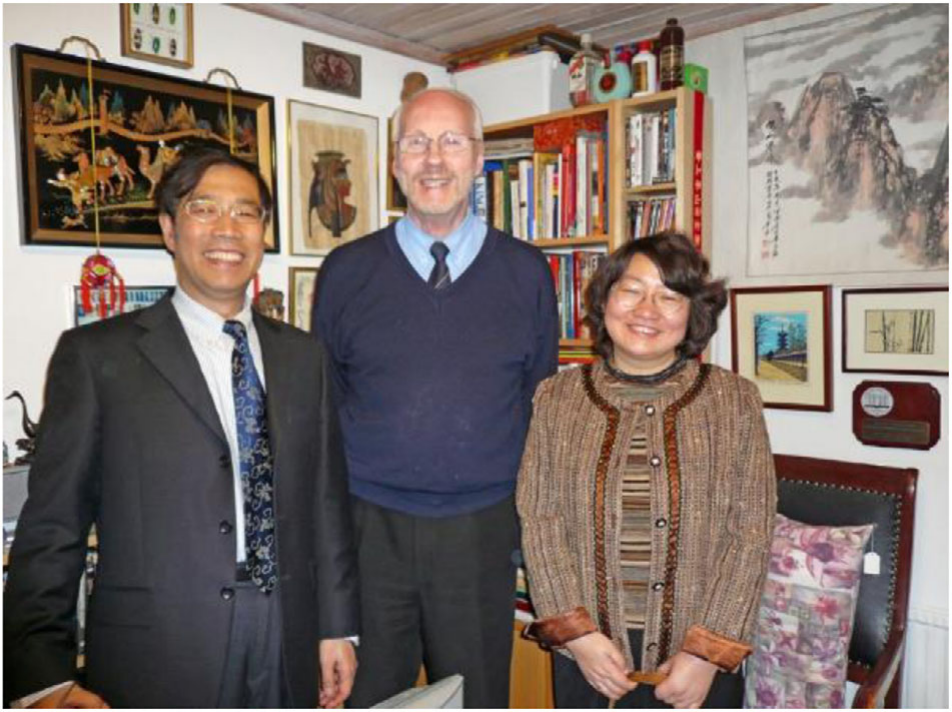
Visit to the home of Prof. Jan‐Christer Janson in Uppsala in 2009 by Prof. Zhiguo Su and Prof. Guanghui Ma. They were invited by GE Healthcare to come to Uppsala for viewing the company's achievement with their technology

Figure 8 is a photo of Prof. Ma and Prof. Su visiting my home. This visit to Uppsala was initiated and organized by the company to show their respect and gratitude to the inventors who helped the company develop new advanced products to the benefit of bioscience and biotechnology. On this occasion Prof. Ma was invited to give a lecture to the company scientists on microsphere preparation engineering.

## 3 COLLABORATIVE INNOVATION ON CHROMATOGRAPHIC PROCESSES

Chromatography is an important technique in the bioscience laboratory as well as in the biotech industry and has been the focus of the collaboration between Prof. Su and me. The reason can be attributed to our knowledge and experience with various protein separation needs, the basis of process science and engineering, and our common interest of innovation.

Apart from chromatographic refolding and chromatographic media design introduced in the previous sections, we have also developed novel chromatographic processes. The first was for the purification of complex and super large vaccine antigens. This purification was previously dominated by the ultracentrifugation technique which is low in resolution and high in operation cost. We have demonstrated the excellent efficacy and resolution of chromatography over conventional techniques in obtaining pure antigens through proper design of the solid phase, the mobile phase and the purification protocols [11, 12]. The second development was on‐line/off‐line characterization and anti‐denaturation strategy using advanced techniques such as multi‐angle laser light scattering, field flow fractionation, and hydrogen deuterium exchange NMR. Discoveries and inventions were made through the combination of process analytical technologies (now termed as PAT). For example, with the increase of ligand density on the solid phase, the contact area between the medium and the complex protein increased which may result in an increased possibility of protein structure change and denaturation [13]. By decreasing the ligand density and modifying the microgeometry of the solid phase, it is possible to preserve the three‐dimensional structure of the protein to ensure a high biological activity.

There would not have been any collaborative success without the substantial input and contributions from Prof. Su. Throughout our collaboration, he has addressed multi‐dimension engineering challenges, taking a unique, creative, systematic but still fundamental approach. The impact of his leadership, not only in research but also in student education and young staff upgrading, has played a key role in the success of chromatography and in the broad area of bioseparation.

Here, I would like to mention the students and staff sent by Prof. Su to Uppsala University for collaborative studies. First in 1999, Mr. Zhenyu Gu, a most able and diligent Ph.D.‐student working on size exclusion chromatography refolding of protein, came to Uppsala University. His work was the first on refolding of protein with process chromatography, and his publications [1, 2] were cited many times by other researchers. Dr. Zhenyu Gu is currently senior process scientist in a US biopharmaceutical company. Next came Ms. Qing Yang, who combined the protein cloning and expression with chromatographic purification for a thrombin‐like enzyme, an important candidate of biomedicine [14, 15, 16]. Dr. Yang is now a distinguished professor of biotechnology at Dalian University of Technology, China. Mr. Ming Li made another important innovation on protein refolding by using ion exchange chromatography (IEC), which has attracted interest not only the academia but also the industrial counterparts because IEC is the most commonly used technique in biotechnology industry [3, 5, 6]. Dr. Li now is a senior engineer in Novozymes, the well‐known Danish biotech company.

After the above successes, we focus on the third commonly used chromatographic technique, hydrophobic interaction chromatography (HIC). Mr. Jingjing Li worked on HIC refolding. He expanded the gradient control to include molecular chaperones to protect the refolding intermediates, and successfully completed the refolding of several pharmaceutical proteins [4, 7, 8]. Dr. Jingjing Li came to the Royal Institute of Stockholm after graduation and later settled in the US working in a biotech research institute.

Among the young visiting scholars from the SKLBCE to Uppsala, Dr. Ming Gu has made outstanding achievements in the development of novel technology for natural product purification. She established a new method for the purification of mussel adhesive protein (MAP), used polysaccharide chromatographic media for the first time to purify a variety of natural products, and published a number of academic papers [17, 18, 19, 20, 21, 22, 23]. Dr. Jian Luo and Dr. Dongxia Hao have also made remarkable progress in the field flow fractionation and hydrogen deuterium exchange NMR studies [13, 24]. All of them, Dr. Gu, Dr. Luo and Dr. Hao, have become project leaders in biotechnology at the Institute of Process Engineering, Chinese Academy of Sciences where the SKLBCE locates.

## 4 THE COLLABORATION WAS HIGHLY ENCOURAGED, RECOMMENDED, AND AWARDED

Sweden and China have a long history of scientific exchange. The collaboration between Prof. Su and me was fortunate to be supported by the scientific and technological agencies on both sides, including the Swedish Biotechnology Development Board (NUTEK later VINNOVA), the Natural Science Foundation of China (NSFC), Uppsala University, and the Institute of Process Engineering, Chinese Academy of Sciences. With these strong supports, I have been able to travel frequently from Sweden to China, while Prof. Su and his colleagues came from China to Uppsala, Sweden.

Through the cooperation, I have made acquaintance with a number of new friends in Chinese universities, research institutions and industries, which has led to a broad spectrum of cooperation, including conference presentation, university lectures, student tutorials, product development, technique trouble shootings, etc.

**FIGURE 9:**
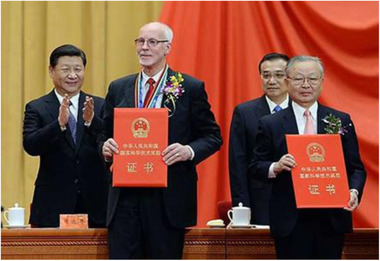
Prof. Jan‐Christer Janson receives the Award by Chairman Xi Jinping in The Great Hall of the People in Beijing on January 8, 2016

My long‐time successful collaboration with my friends in China had brought a great honour to me. Thus, in 2016, I was awarded the “International Scientific and Technological Cooperation Award of the Peoples Republic of China” (Figure 9) that is very prestigious. The nomination was initiated by the Chinese Academy of Sciences in which Prof. Guanghui Ma was the team leader for the recommendation, supported by Prof. Zhiguo Su, Prof. Zheng Liu (Tsinghua University), and Prof. Tianwei Tan (Beijing University of Chemical Technology).

Finally, I would like to express my sincere thanks to Prof. Su for his long‐time cooperation, hospitality, friendship, and support. He certainly deserves a special issue of *Engineering in Life Science* because he has been an influential leader of biochemical engineering in China with innovative research and educational initiatives, both nationally and internationally. He has inspired the young biochemical engineers in his country to pursue the translation of theoretical investigation into applicational successes, a doctrine he set up for the SKLBCE. As you can see from this special issue, innovative research developments are reported and presented, which reflect the advancement of biochemical engineering activities by Prof. Su's colleagues, friends, and students. It is a remarkable salute to Professor Zhiguo Su.
